# Professional interaction in management of the triad: Permanent Education in Health, patient safety and quality*

**DOI:** 10.1590/1518-8345.4154.3379

**Published:** 2020-09-30

**Authors:** Cintia Koerich, Alacoque Lorenzini Erdmann, Gabriela Marcellino de Melo Lanzoni

**Affiliations:** 1 Secretaria de Estado da Administração de Santa Catarina, Diretoria de Saúde do Servidor, Florianópolis, SC, Brazil.; 2 Universidade Federal de Santa Catarina, Departamento de Enfermagem, Florianópolis, SC, Brazil.

**Keywords:** Continuing Education, Patient Safety, Quality Management, Interdisciplinarity, Health Management, Nursing, Educação Continuada, Segurança do Paciente, Gestão da Qualidade, Interdisciplinaridade, Gestão em Saúde, Enfermagem, Educación Continua, Seguridad del Paciente, Gestión de la Calidad, Interdisciplinariedad, Gestión en Salud, Enfermería

## Abstract

**Objective:**

to understand how professional interaction takes place in the hospital organizational structure for the management of Permanent Education in Health, to guarantee patient safety and the quality of nursing care.

**Method:**

this is a qualitative study, which used the structuralist aspect of the Grounded Theory as a methodological framework. 27 interviewers participated in the study, who made up four sample groups.

**Results:**

six categories and 13 subcategories were presented, representing the studied phenomenon and highlighting particularities of the public health system and the influence of the manager’s support and management priority, the disposition of the organizational structure, the institutional culture, the external encouragement to institution, and the nurses’ initiative and leadership in the professional interaction for the management of the Permanent Education in Health, patient safety, and quality of care triad, revealing the need for cultural change through interdisciplinarity.

**Conclusion:**

the professional interaction in the hospital organizational structure requires the creation of new management models with an emphasis on more participative management, in order to improve the care processes in hospital institutions.

## Abstract (pt)

**Objetivo:**

compreender como acontece a interação profissional na estrutura organizacional hospitalar para a gestão da educação permanente em saúde a fim de garantir a segurança do paciente e a qualidade do cuidado de enfermagem.

**Método:**

trata-se de um estudo qualitativo, que utilizou a vertente estruturalista da Teoria Fundamentada em Dados como referencial metodológico. Participaram do estudo 27 entrevistados, que compuseram quatro grupos amostrais.

**Resultados:**

apresentaram-se seis categorias e 13 subcategorias que representam o fenômeno estudado e destacam particularidades do sistema público de saúde, a influência do apoio do gestor e da prioridade de gestão, da disposição da estrutura organizacional, da cultura institucional, dos estímulos externos à instituição, da iniciativa e da liderança do enfermeiro na interação profissional para a gestão da tríade Educação Permanente em Saúde, segurança do paciente e qualidade da assistência, revelando a necessidade de uma mudança cultural por meio da interdisciplinaridade.

**Conclusão:**

a interação profissional na estrutura organizacional hospitalar exige a conformação de novos modelos de gestão com ênfase em uma gestão mais participativa, de forma a melhorar os processos de cuidado nas instituições hospitalares.

## Abstract (es)

**Objetivo:**

comprender cómo se produce la interacción profesional en la estructura organizativa hospitalaria para la gestión de la educación permanente en salud a fin de garantizar la seguridad del paciente y la calidad de la atención de enfermería.

**Método:**

se trata de un estudio cualitativo, que utilizó el aspecto estructuralista de la Teoría Basada en Datos como referencia metodológica. Participaron en el estudio 27 entrevistados, que conformaron cuatro grupos de muestra.

**Resultados:**

se presentaron seis categorías y 13 subcategorías que representan el fenómeno estudiado y destacan particularidades del sistema público de salud, la influencia del apoyo del gerente y la prioridad de la gestión, la disposición de la estructura organizativa, la cultura institucional, los estímulos externos a la institución, la iniciativa y el liderazgo del enfermero en la interacción profesional para la gestión de la tríada de Educación Permanente en Salud, seguridad del paciente y calidad de la atención, revelando así la necesidad de un cambio cultural a través de la interdisciplinariedad

**Conclusión:**

la interacción profesional en la estructura organizativa hospitalaria requiere la conformación de nuevos modelos de gestión con énfasis en una gestión más participativa, para mejorar los procesos asistenciales en las instituciones hospitalarias.

## Introduction

The Health Care Network (*Rede de Atenção à Saúde*, RAS) in Brazil proposes an integration among services and aims to contemplate the doctrinal and organizational principles of the Unified Health System (*Sistema* Único *de Saúde*, SUS). However, the system still faces obstacles to consolidate this integration, and the fragmentation is also reflected within hospital institutions with isolated and incommunicable departments, which directly affects the resolvability, accountability, and integrality of care^(^ 1 ^)^.

The organizational structure of Brazilian public hospitals usually follows the guidelines established by the classic organization charts of the administration, in which hierarchy, authoritarianism, and decision-making centrality stand out, which results in little cooperation, difficulty in working in teams and developing interdisciplinarity^(^ 2 ^)^.

In this sense, considering the history of fragmentation of hospital institutions, with regard to the arrangement of the organizational structure, as well as the performance of health professionals, it is relevant to investigate how these aspects affect the management of Permanent Education in Health (PEH) for patient safety and quality, since education is an essential practice in building safer and quality patient care^(^ 3 ^-^ 4 ^)^. Furthermore, it can be said that the quality of hospital care is directly related to the quality of care provided by the professionals who work in this scenario.

A number of studies discuss patient safety and quality of care as if they were interdependent elements. However, PEH is considered an important, but still secondary, component in the context of the implementation of safety and quality actions in hospital institutions^(^ 3 ^-^ 5 ^)^. This study defends the need to place these three axes in the same level of importance and relevance in order to achieve the articulation indispensable to the necessary paradigmatic change.

Thus, the management of PEH in hospital institutions carries the need for interaction between the actors that make up this scenario, such as managers, professionals, and patients, considering that human interaction promotes the construction of new knowledge or the reformulation of other knowledge previously evidenced, and that health care demands the permanent articulation of knowledge and professional practices in order to build a more comprehensive health model^(^ 6 ^)^.

In this scenario, nurses are important actors in the process of transition and restructuring of the services, since they have management training and are constantly involved in actions that encompass the inclusion of patients in health care, the improvement of the nursing team, the integration of the multiprofessional team, humanization of care, patient safety, and quality of care, among others.

In this sense, the following question emerges: How does the professional interaction take place in the hospital organizational structure for management of Permanent Education in Health in order to ensure patient safety and the quality of nursing care?

The study aimed to understand how professional interaction takes place in the hospital organizational structure for the management of Permanent Education in Health in order to ensure patient safety and the quality of nursing care.

## Method

This is a qualitative study, which used the structuralist aspect of the Grounded Theory (GT) as a methodological framework.

The scenario selected included hospital institutions under direct public administration in a state in southern Brazil. Among the 13 institutions, three were chosen as the collection scenario, all being large hospitals located in three different regions of the state, as well as the State Health Secretariat (SHS), which were included in the study according to the theoretical sample^(^ 7 ^)^.

The participants were intentionally chosen according to the objective of the study and approached in their own work environment. The inclusion criterion was that the participant had a minimum experience of one year at the institution and was involved with the study theme. 27 participants were interviewed, among sector chiefs (professionals responsible for in-hospital care units/professionals working in them with greater involvement in the theme), coordinators, managers and directors at local (hospital) and state (SHS) levels.

As for the participants’ training, 22 were nurses and 5 had other graduation degrees (2 physical educators, 1 lawyer, 1 designer and 1 pharmacist). As regards gender, 24 were female and 03, male. Their ages ranged between 30 and 55 years old (14 participants were aged between 30 and 40, 9 between 41 and 50, and 4 were over 50 years old). Their length of service varied between 5 and 25 years (13 participants had between 5 and 10 years of service, 12 had between 11 and 20 years, and 2 had over 20 years).

The participants were organized into four sample groups guided by the questions and hypotheses raised after analyzing the data, as shown in Figure 1.

**Figure 1:** Theoretical sampling presentation

*Sample Group*	*Number of Participants*	*Questions*	*Hypothesis Raised*
First SG* Hospital A	08 participants: 04 unit chiefs, 01 manager, 04 support services coordinators	Tell me about how PEH^†^ practices are managed at this institution.	In hospitals where the management prioritizes patient safety and quality of care, the interaction among the professionals for the management of PEH^†^ tends to be boosted.
Second SG* Hospital B	07 participants: 03 unit chiefs, 01 manager, 04 support services coordinators	Tell me about the management's performance in managing PEH^†^, patient safety, and quality of care in the institution.	In addition to management support, projects with shared goals at the institutional and extra-institutional levels enhance professional interaction in the management of PEH^†^ with a focus on patient safety and quality of care.
Third SG* Hospital C	08 participants: 02 unit chiefs, 01 manager, 05 support services coordinators	Tell me about the PEH management^†^, patient safety, and quality of care interface with initiatives and projects that are being implemented at the institution.	The SHS^‡^ offers autonomy to hospital institutions for managing goals related to PEH^†^, to patient safety, and to quality of care in order to favor professional interaction and cultural changes.
Fourth SG* SHS	04 participants: 01 director, 03 coordinators	Tell me about the role of the SHS^‡^ in managing PEH^†^, patient safety, and quality of care in hospital institutions.	

* SG = Sample group;

† PEH = Permanent education in health;

‡ SHS = State Health Secretariat

The interviews were recorded in digital audio, after signing the Free and Informed Consent Form (FICF), between June and December 2018. These started with a guiding question, in order to give the participants freedom to express themselves on the theme: *Talk to me about how the PEH practices are managed in this institution.* Then, other questions were made to the participants to explore the meanings through an in-depth interview until reaching theoretical saturation^(^ 7 ^-^ 8 ^)^.

Data analysis took place by means of three interdependent stages called open coding, axial coding, and integration. The results were presented in the paradigm composed of the following components: condition; action-interaction, and consequence^(^ 7 ^)^. Data was organized using the NVIVO^®^ software, version 12. The theory was validated at a meeting of the Patient Safety Committee (*Comitê de Segurança do Paciente*, COSEP) at the SHS, attended by six nurses with expertise in the study theme^(^ 7 ^,^ 9 ^)^.

The research observed Resolution 466/2012 and was approved under CAAE 80479717.2.0000.0121.

## Results

**Figure 2:**
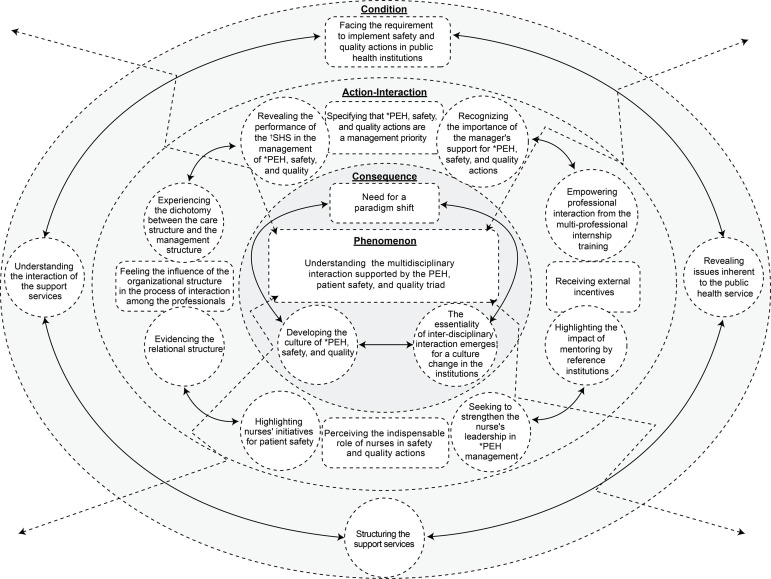
Integrative diagram of the phenomenon Giving meaning to the complexity of the professional interactions in the management of PEH, patient safety, and quality in public hospitals PEH = Permanent education in health; †SHS = State Health Secretariat

The results show the central category of *Understanding the multiprofessional interaction supported by the PEH, patient safety, and quality triad*, which lists seven categories and 13 subcategories that were organized by using the components of the paradigm composing the substantive theory. This was represented in Figure 2 by the integrative diagram of the phenomenon *Giving meaning to the complexity of the professional interactions in the management of PEH, patient safety, and quality in public hospitals*.

The central category reveals that the PEH, safety, and quality triad is the guiding line of the professional interaction process in hospital institutions. PEH management was not revealed in isolation, since PEH, patient safety, and quality of care showed a movement of interdependence. In this context, the professional interaction reveals the concern with improving patient care, which means a need to work for safe and qualified care. In turn, this care refers not only to nursing care, but to multiprofessional and interdisciplinary health care centered on the patient, this configuration being essential in the process of breaking the culture of fragmentation in hospital institutions.

The *Condition* component was represented by the category of *Facing the requirement to implement safety and quality actions in public health institutions*, which is composed of three subcategories.

The first subcategory, *Revealing issues inherent to the public health service*, highlights management difficulties in the public health services that impact on safety and quality actions. Among them, the high turnover of management positions, the managers’ lack of training and experience in the management field, inadequate management of material and human resources, and discontinuity of projects.

The second subcategory, *Structuring the support services*, presents the organization of the support services given the need to implement patient safety actions in hospital institutions, in response to patient safety legislation. The following were considered as support services: the Center for Permanent Education (*Núcleo de Educação Permanente*, NEP) in Health, the Patient Safety Center (*Núcleo de Segurança do Paciente*, NSP), and the Quality Center (*Núcleo de Qualidade*, NQ), among others, which, within the organizational structure, aim to support the professionals regarding safety and quality in patient care. It was observed that hospitals have different organizational structures for the provision of these services, but sharing the same physical space contributes to the sharing of information, responsibilities, and joint actions.

The third subcategory, *Understanding the interaction of the support services*, shows how the coordinators of the support services sought to raise the awareness of care professionals *in loco* to implement safety and quality actions, aiming at breaking an institutionalized model of fragmented work in favor of a dynamic and proactive work. Such actions were considered to be PEH practices, since they were carried out within an institutional movement to improve patient safety and quality of care. The NSP was also identified as an important meeting and interaction space among the representatives of the support services and the multiprofessional team.

The *Action-interaction* component was represented by four categories and their respective subcategories. The first category, *Feeling the influence of the organizational structure in the process of interaction among the professionals*, is composed of two subcategories. The first subcategory, *Experiencing the dichotomy between the care structure and the management structure*, reveals how the organizational structure of the hospitals interferes with the professional relationships and interactions. The fragmentation between care and management structures in the hospitals was considered a cultural issue since, culturally, an administrative position is more valued than a care position, given the institutional hierarchy. This gap between the support services and the care units results in low adherence to reporting adverse events and PEH practices, as well as difficulty in implementing patient safety protocols.

The second subcategory, *Evidencing the relational structure*, shows that interpersonal relationships need to be a concern for managers so that the management of PEH, safety, and quality takes place in the practice. Miscommunication was considered a deficiency in public hospitals, an important obstacle to professional interaction, considering the ideological and behavioral differences among the professionals. Thus, the need for spaces of multiprofessional interaction was emphasized in order to favor interpersonal relationships.

The second category, *Specifying that PEH, safety, and quality actions are a management priority*, presents *Recognizing the importance of the manager’s support for PEH, safety, and quality actions* as its first sub-category, which addresses the need for planning, participation, supervision, support and demands from the managers, especially hospital directors, regarding the implementation of actions and projects in hospital institutions. The management’s support for improvement actions is essentially conditioned to understanding the importance of the support services. These services were considered to be lacking managerial support given the low structure, insufficient human resources, and distance from the management. The participants report the need for shared management in order to modify a reality in which the decision process is centralized in the management.

The second subcategory, *Unveiling the role of the SHS in the management of PEH, safety, and quality*, presents the SHS initiatives aimed at quality of care and patient safety in hospitals. Although SHS has the legitimacy to manage hospital institutions, there is a tendency to give autonomy to the own institutions’ managements in comparison to their internal organization. The participants highlight the need for the SHS to closely monitor the development of actions and projects that occur within the hospitals.

The third category, *Receiving external encouragement*, is composed of two subcategories. The first subcategory, *Empowering professional interaction from the multiprofessional internship training*, describes how the multiprofessional internship training contributes to the interaction among the professionals in the management of PEH, safety, and quality. The impact of the multiprofessional internship training proved to be relevant for considerably reducing barriers between the disciplines and having modified professional relationships, contributing to interdisciplinary interaction, and improving the articulation between theory and practice, as well as in the assistance itself, which enhanced the service and the quality of the discussion among the professionals.

The second subcategory, *Highlighting the impact of mentoring by reference institutions*, reveals how ministerial initiatives, such as the Institutional Development Support Program of the SUS (*Programa de Apoio ao Desenvolvimento Institucional do SUS*, PROADI-SUS), are able to favor interaction among the professionals in hospital institutions in the management of PEH, safety, and quality, considering that since the beginning they require active participation of the management, as well as of the multiprofessional team. Through the projects entitled “Improving patient safety at a large scale in Brazil” and “*Lean* in the emergency”, it was noticed that the presence of representatives of institutions (that are a reference in the country) in the hospitals increased the professionals’ adherence and commitment to the proposed actions.

The fourth category, *Perceiving the indispensable role of nurses in safety and quality actions*, presents the first subcategory, *Highlighting nurses’ initiatives for patient safety*, which reveals the nurses’ participation in actions and projects aimed at patient safety in the hospitals. The participants state that the nursing team acquired greater awareness, adhering to protocols and extra-institutional projects, in relation to other health professionals. Thus, nurses were considered managers of patient safety protocols, since they are usually in charge of the support services, commissions, actions, and decision-making, although the need for involvement and participation of the multiprofessional team was indicated.

The second subcategory, *Seeking to strengthen the nurse’s leadership in PEH management*, highlights the main aspects to be addressed in order to develop in nurses the essential professional leadership for the management of PEH, safety, and quality in the hospitals. The nurses’ leadership is highlighted, since they have a fundamental role in managing the nursing team. Thus, reflection, encouraging reflection in the team, meetings for the discussion, and supervision of the work process are important actions that require leadership from nurses, considering that the insertion of the technical team in the discussions showed positive results in terms of adherence to the change processes.

The *Consequence* component is represented by the *Need for a paradigm shift* category, which has two subcategories. The first subcategory, *Developing the culture of PEH, safety, and quality*, presents the need to build a new institutional culture in view of the current configuration in the hospital scenario, which makes it difficult for PEH, safety, and quality actions to become effective.

The need for accountability, planning, and multiprofessional and interdisciplinary PEH actions was highlighted for building the PEH culture in hospital institutions. Furthermore, the challenges in the professionals’ adherence to the basic patient safety protocols and to the notification of adverse events, as well as to the incipient risk management, were indications of the absence of a safety culture. The need for standardization, analysis of indicators, implementation of preventive actions, and few actions focused on the patient were also considered indicative of the absence of a quality culture.

The second subcategory, *The essentiality of interdisciplinary interaction emerges for a culture change in the institutions*, shows the need to break the culture of fragmentation present in hospital institutions and to create a new culture through interdisciplinary interaction. The existence of “two worlds”: the Medicine world and the world of other professions, that is, the overvaluation of one profession to the prejudice of others in the hospital setting is responsible for interpersonal conflicts and professional demotivation. According to the participants, the fragmentation of work processes originates from professional training, considering the lack of interaction among the disciplines, as well as the development of the professionals in different logics within the institutions, which reinforces disciplinary fragmentation.

However, despite the confrontations, some initiatives were mentioned as enhancing the interdisciplinary interaction, such as the unique therapeutic project, the standardization of the health practices by means of protocols, Standard Operating Procedures (SOP) or multidisciplinary multiprofessional checklists, the implementation of instruments to improve multiprofessional communication such as *SBAR*, *Kanban* and the *Rounds,* as well as formal spaces for interaction.

## Discussion

The first point to be discussed (which has a strong impact when talking about the management of Permanent Education in Health, patient safety, and quality of care in public hospitals) is the instability and high turnover of managers in this scenario, which results in discontinuity of projects, constant restarts, and consequent demotivation of the professionals^(^ 1 ^,^ 10 ^)^. In addition to the frequent change of managers, the scarcity of professionals prepared to work in the management of the SUS is a reality and was linked in a study to the *deficit* in the management training of health professionals and to inefficient permanent education in the field. Furthermore, the manager’s difficulty in seeking management models beyond the traditional one, characterized by centralized, hierarchical, and bureaucratic actions, represents the managerial ability incompatible with the needs and complexities of the health care sector^(^ 1 ^)^.

In order to integrate and enhance safety and quality actions, in some hospitals of this study the support services started to take place in the same physical space. However, the need was clear for a change in the mentality of the professionals to understand that this work requires more than sharing the same physical space, being necessary to work in an integrated way. Thus, the articulation between the different services in the organizational structure is conditioned to the recognition of the insufficiency of these isolated services in providing comprehensive care to the patient^(^ 6 ^)^.

PEH management was identified when, aiming to implement safety and quality actions, the coordinators of the support services sought to approach and raise awareness among care professionals through *in loco* educational actions. These educational actions, when carried out within an institutional plan or project, such as the patient safety plan and certification project, can be characterized as PEH practices, as they have a continuity character, undergo an evaluation process, and meet organizational objectives^(^ 11 ^)^.

This context, however, requires the encouragement of effective communication, feedback to the professionals, management support, teamwork, autonomy, and professional initiatives, these being potential actions to reduce resistance to change by the professionals, in addition to contributing to innovation in the health care services^(^ 12 ^)^.

The organizational structure interferes with the management of the triad because it is directly influenced by the organizational culture, which can be understood as a process that involves a combination of factors such as customs, habits, rules, and formal and informal communication, among others^(^ 13 ^-^ 14 ^)^ that influence all actions within the institution. This is partly the result of the training of the health professionals, since it occurs in a fragmented way and ends up building strict professional identities, which hinders communication and interaction among them^(^ 15 ^)^. As well as the mentality and individual cultures established at each level of the organization, interfering with the implementation of improvements^(^ 16 ^)^.

This structure was characterized in this study by a strong dichotomy between the care dimension and the management dimension, which ends up interfering with the participation and interaction of the professionals in the change processes. A number of studies indicate that this situation causes concerns among nurses, as it directly and negatively influences their work process^(^ 2 ^,^ 17 ^)^.

Another issue concerns the relational structure, that is, how these professionals usually relate within the organizational structure. In this sense, it is necessary to consider that the emphasis on the relational dimension and interpersonal relationships by the managers reinforces the orientation that the services are made up of people and that they are essential in all the processes^(^ 16 ^)^. To this end, the institutions must have collegiate spaces, such as centers and commissions, which can be daily shared by the professionals in order to favor interaction^(^ 18 ^)^, communication^(^ 19 ^-^ 21 ^)^, and teamwork^(^ 18 ^)^.

The managers’ support was revealed in this study as an important encouragement in the implementation of institutional improvement processes since, even though they are not the only ones responsible for the success of the institutional projects, they are responsible for strengthening the interaction of the multiprofessional team, monitoring and demand in order to attain the institutional objectives. Several studies reinforce the support of the manager as paramount in hospital institutions, either for the development of professional skills^(^ 22 ^)^, of the culture of safety and institutional performance^(^ 3 ^,^ 12 ^,^ 19 ^,^ 23 ^)^ or of the effectiveness of PEH^(^ 24 ^)^.

In addition, despite the results presenting SHS initiatives to support the triad management in the hospital institutions, the participants reinforced the need for the SHS to monitor and demand actions. In this sense, there is a need for “participative supervision” that helps, empowers, and enables active and critical participation in order to develop professional skills with the potential to transform reality^(^ 25 ^)^.

Extra-institutional incentives, such as multiprofessional internship training and PROADI-SUS projects, were considered to be boosters of the triad management in this study, as they motivate interdisciplinary interaction. They represent the articulation of the public service as a private sector and with teaching/training. The multiprofessional internship training has the potential to change the health professionals’ attitude, sensitizing them to act beyond the current paradigms, being able to provide the integration of different disciplines by articulating specific knowledge and sharing actions^(^ 26 ^)^.

In the implementation and development of the projects coordinated by PROADI-SUS, it was noticed that the professionals’ satisfaction and motivation were directly related to their participation in the decision-making process. A study carried out at the Valladolid Hospital in Spain assessed the nursing staff’s level of satisfaction with the implementation of measures to improve the quality of care and showed that the staff’s satisfaction favored patient safety and satisfaction in addition to quality of care^(^ 27 ^)^, corroborating the results of this study.

Nurses stood out as the protagonists of safety and quality actions for their initiatives in the hospital scenario; however, there is a need to strengthen their leadership ability given the importance of their role, especially when related to the management of PEH. The practice of leadership is associated with positive results in the health scenario, such as team satisfaction and engagement, quality of care, and construction of the organizational culture^(^ 28 ^)^. In addition, committed leadership, effective communication, feedback, and environments focused on learning were considered in the study as potential factors for improving institutional processes^(^ 21 ^)^.

A study showed that the nursing staff has a better understanding, perception, and attitude towards patient safety when compared to other health professionals^(^ 23 ^)^. However, it should be noted that the fact that nurses are more engaged in safety actions does not place them as the main responsible for the success or not of these actions at the institutional level. Building a safety culture is a joint work that involves the multiprofessional team and managers.

Finally, the results show the need for a paradigm shift based on the construction of the culture of PEH, safety, and quality supported by interdisciplinarity. Interdisciplinary interaction is one of the main challenges for health care. The professionals need to learn to work in an integrated way, to break the disciplinary barriers, and to rethink ways of interaction so that health care is integrative^(^ 29 ^)^, since the fragmentation of the work process hinders work in interdisciplinary team^(^ 2 ^)^ and interferes with the construction of a new culture^(^ 21 ^)^.

In this regard, it is worth noting that cultural change in an institution is not a radical replacement of an old model with a new one, but rather a construction process that involves tensions and conflicts since, in the hospital scenario, the current model of health care, focused on diseases and fragmented work, lives with the perception of the need for interdisciplinary work, which questions the fragmentation of the care provided to the patient^(^ 30 ^)^.

Interdisciplinary interaction means a relationship of interdependence and cooperation between different disciplines around a common goal^(^ 31 ^)^ and, therefore, it is necessary to resist biomedical logic^(^ 32 ^)^. Power disputes hinder the implementation of collegiate spaces, as the interests of certain professional categories have a strong influence on the management of the hospitals, resulting in resistance to maintain power and, consequently, hindering change processes^(^ 30 ^)^.

Thus, in order to favor interdisciplinary interaction in the hospitals, unified protocols can facilitate the process of information and communication among the professionals, in addition to promoting patient safety and supporting PEH actions to implement quality care^(^ 27 ^)^. In this sense, instruments like *Kanban* ^(^ 33 ^)^ and SBAR^(^ 34 ^)^ are worth mentioning.

Furthermore, initiatives such as the unique therapeutic project offer opportunities for new ways to conduct the practice, work as a team, and co-manage^(^ 35 ^)^. A proposal similar to the therapeutic project was presented in a study carried out in the United States of America, in which hospitals have been investing in new ways of providing care to include other perspectives in a single care plan^(^ 36 ^)^.

In short, the theory presented reveals that the professional interactions in the management of PEH, patient safety, and quality in public hospitals is a complex phenomenon supported by the triad of Permanent Education in Health, safety, and quality, which is presented as an articulated and interrelated basis. This interaction is enhanced by the priority of management, internal initiatives, and extra-institutional encouragement, and is conditioned to a paradigm shift, whether in the way of thinking and doing health, or in the way of restructuring the work processes in hospital institutions.

The theory identifies the main challenges and institutional initiatives to manage processes to improve public hospital services and sees the importance of managing the triad of PEH, safety, and quality in an integrated manner and with the interaction of different actors working internally, in an interdisciplinary way, and externally to the hospital, in order to obtain different health outcomes.

The study presented as a limitation the fact that it was targeted to managers and, therefore, it did not include all the professionals of the multiprofessional team. However, it has the potential to assist managers in the identification of the critical nodes, as well as in the development of strategies for the management of the PEH, patient safety, and quality triad in public hospitals.

## Conclusion

The results reveal that the professional interactions in the hospital setting were motivated by the PEH, safety, and quality triad. In this scenario, the management of this triad is permeated by issues inherent to the public health service, such as frequent changes of managers, management priorities, disposition of the organizational structure, fragmentation of the work process, devaluation of the support services, and occasional and unprofessional educational practices, which have an impact on motivation, satisfaction, and adherence to change processes.

As enhancers of multiprofessional and interdisciplinary interaction, initiatives such as the implementation of the multiprofessional internship training and of extra-institutional projects were highlighted, as well as the encouragement of the role of nurses through initiatives and leadership development with support from the SHS.

There was a need for an emphasis on interpersonal relationships, collegiate spaces, management support, monitoring of *in loco* actions and a paradigm shift based on the construction of the PEH, safety, and quality culture through interdisciplinarity. This configuration requires new management models with an emphasis on a more participative management, which is based on the interaction of the professionals in a horizontal and democratic way, contributing to improve the care processes in the hospital institutions and for this care to be targeted to users’ needs.

## References

[1] Lorenzetti J, Lanzoni GMM, Assuiti LFC, Pires DEP, Ramos FRS (2014). Health management in Brazil: dialogue with public and private
managers. Texto Contexto Enferm.

[2] Rocha FLR, Marziale MHP, Carvalho MC, Cardeal IFS, Campos MCT (2014). The organizational culture of a Brazilian public
hospital. Rev Esc Enferm USP.

[3] Alzahrani N, Jones R, Abdel-Latif ME (2018). Attitudes of doctors and nurses toward patient safety within
emergency departments of two Saudi Arabian hospitals. BMC.

[4] Olds DM, Aiken LH, Cimiotti JP, Lake ET (2017). Association of nurse work environment and safety climate on
patient mortality: A cross-sectional study. Int J Nurs Stud.

[5] Ramírez E, Martín A, Villán Y, Lorente M, Ojeda J, Moro M (2018). Effectiveness and limitations of an incident-reporting system
analyzed by local clinical safety leaders in a tertiary hospital.
Prospective evaluation through real-time observations of patient safety
incidents. Medicine.

[6] Reeves S, Xyrichis A, Zwarenstein M (2018). Teamwork, collaboration, coordination, and networking: why we
need to distinguish between different types of interprofessional
practice. J Interprof Care.

[7] Corbin J, Strauss A (2015). Basics of Qualitative research: Techniques and procedures for developing
Ground Theory.

[8] Strauss A, Corbin J (2008). Pesquisa qualitativa: técnicas e procedimentos para o desenvolvimento de
teoria fundamentada. 2 ed.

[9] Glaser B, Strauss A (1967). The Discovery of grounded theory.

[10] Reis GAX, Hayakama LY, Murassaki ACY, Matsuda LM, Gabriel CS, Oliveira MLF (2017). Nurse manager perceptions of patient safety strategy
implementation. Texto Contexto Enferm.

[11] Koerich C, Erdmann AL (2016). Managing educational practices for qualified nursing care in
cardiology. Rev Bras Enferm.

[12] Berberoglu A (2018). Impact of organizational climate on organizational commitment and
perceived organizational performance: empirical evidence from public
hospitals. BMC.

[13] Marulanda C, Lopez L, Cruz G (2018). La Cultura Organizacional, Factor Clave para la Transferencia de
Conocimiento em los Centros de Investigación del Triángulo del Café de
Colombia. Inf. Tecnol.

[14] Klimas P (2016). Organizational culture and competition: An exploratory study of
the features, models and role in the Polish Aviation
Industry. Ind Mark Manag.

[15] Weller J, Boyd M, Cumin D (2014). Teams, tribes and patient safety: overcoming barriers to
effective teamwork in healthcare. Postgrad Med J.

[16] Lee J-C, Shiue Y-C, Chen C-Y (2016). Examining the impacts of organizational culture and top
management support of knowledge sharing on the success of software process
improvement. CBH.

[17] Treviso P, Peres SC, Silva AD, Santos AA (2017). Competências do enfermeiro na gestão do cuidado. Rev Adm Saúde.

[18] Lavich CRP, Terra MG, Mello AL, Raddatz M, Arnemann CT (2017). Permanent education actions of nurse facilitators at a nursing
education centre. Rev Gaúcha Enferm.

[19] Cavalcante EFO, Pereira IRBO, Leite MJVF, Santos AMD, Cavalcante CAA (2019). Implementation of patient safety centers and the
healthcare-associated infections. Rev. Gaúcha Enferm.

[20] Najjar S, Baillien E, Vanhaecht K, Hamdan M, Euwema M, Vleugels A (2018). Similarities and differences in the associations between the
dimensions of patient safety culture and self-reported outcomes in two
different cultural contexts: a national cross-sectional study in Palestinian
and Belgian hospitals. BMJ.

[21] Okuyama JHH, Galvao TF, Silva MT (2018). Healthcare Professional's Perception of Patient Safety Measured
by the Hospital Survey on Patient Safety Culture: A Systematic Review and
Meta-Analysis. ScientificWorldJournal.

[22] Toles M, Colón-Emeric C, Naylor MD, Barroso J, Anderson RA (2016). Transitional care in skilled nursing facilities: a multiple case
study. BMC.

[23] Carvalho REFL, Arruda LP, Nascimento NKPN, Sampaio RL, Cavalcante MLSN, Costa ACP (2017). Assessment of the culture of safety in public hospitals in
Brazil. Rev. Latino-Am. Enfermagem.

[24] Campos KFC, Sena RR, Silva KL (2017). Permanent professional education in healthcare
services. Esc Anna Nery.

[25] Hoffmann LMA, Koifman L (2013). O olhar supervisivo na perspectiva da ativação de processos de
mudança. Physis.

[26] Silva CT, Terra MG, Kruse MHL, Camponogara S, Xavier MS (2016). Multi-professional residency as an intercessor for continuing
education in health. Texto Contexto Enferm.

[27] Cidón EU, Martín FC, Villaizán MH, Lara LF (2012). A pilot study of satisfaction in oncology nursing care: an
indirect predictor of quality of care. Int J Health Care.

[28] West M, Armit K, Loewenthal L, Eckert R, West T, Lee A (2015). Leadership and Leadership Development in Health Care: The Evidence
Base..

[29] Gomes IEM, Signor E, Arboit EL, Colomé ICS, Silva LAA, Correa AMG (2014). Desafios na gestão do trabalho em saúde: a educação na interface
com atenção. Rev Enferm. Centro Oeste Mineiro.

[30] Silva AM, Sá MC, Miranda L (2015). "Fiefdoms" and co-management: the paradox of autonomy in an
experience of democratization of hospital management. Ciênc Saúde Coletiva.

[31] Costa MV, Peduzzi M, Freire JR, Silva CBG (2018). Educação Interprofissional em Saúde..

[32] Ribeiro ACL, Ferla AA (2016). Como médicos se tornaram deuses: reflexões acerca do poder médico
na atualidade. Psicol Rev.

[33] Massaro IAC, Massaro A (2017). O Uso do KANBAN na Gestão do Cuidado: Superando
Limites. Rev Adm Saúde.

[34] Raymond M, Harrison MC (2014). The structured communication tool SBAR (Situation, Background,
Assessment and Recommendation) improves communication in
neonatology. S Afr Med J.

[35] Lima SAV, Albuquerque PC, Wenceslau LD (2014). Educação permanente em saúde segundo os profissionais da gestão
de Recife, Pernambuco. Trabalho, Educação e Saúde.

[36] Gonzalo JD, Himes J, Mcgillen B, Shifflet V, Lehman E (2016). Interprofessional collaborative care characteristics and the
occurrence of bedside interprofessional rounds: a cross-sectional
analysis. BMC.

